# Sensory Sensitivity and Intolerance of Uncertainty Influence Anxiety in Autistic Adults

**DOI:** 10.3389/fpsyg.2021.731753

**Published:** 2021-11-16

**Authors:** Karys M. Normansell-Mossa, D. Nicholas Top, Nicholas Russell, Mark Freeston, Jacqui Rodgers, Mikle South

**Affiliations:** ^1^Department of Psychology, Brigham Young University, Provo, UT, United States; ^2^Student Health Services, Utah Valley University, Orem, UT, United States; ^3^School of Psychology, Newcastle University, Newcastle upon Tyne, United Kingdom; ^4^Population Health Sciences Institute, Sir James Spence Institute, Newcastle upon Tyne, United Kingdom; ^5^Neuroscience Center, Brigham Young University, Provo, UT, United States

**Keywords:** autism, sensory sensitivity, intolerance of uncertainty, anxiety, autistic adults, SS, IUC

## Abstract

Several models of anxiety in autistic adults have focused on the role of intolerance of uncertainty which has biological and evolutionary bases, as a cognitive explanation for the high prevalence of anxiety in autism. This framework suggests that all people are born with a healthy level of intolerance of uncertainty, and as we develop, this intolerance is lessened as we learn when situations are safe and begin to understand and manage the uncertainty. This process of learning about managing uncertainty does not happen in the same way in those who are high in autistic traits, which could be the reason for the high levels of anxiety symptoms commonly seen in this population. We examined archival data of 199 non-autistic and 55 autistic adults from prior studies in which we collected self-report measures of autistic traits, intolerance of uncertainty, sensory processing, and anxiety. We conducted two path analyses to examine the role of intolerance of uncertainty in anxiety in autistic adults. The first model tested the idea that intolerance of uncertainty, an evolutionary phenomenon common for all people, could explain some of the cognitive aspects of anxiety in autism. The second model suggests that primary neurodevelopmental differences associated with autistic traits underlie the sensory sensitivity and sensory seeking behaviors, which in turn increase intolerance of uncertainty and subsequent anxiety. We found that the “neurodevelopmental” model had better model fit than the “evolutionary stress” model, suggesting that the neurodevelopmental impact of higher levels of autistic traits could moderate a non-autistic trajectory of learning to manage uncertainty as children develop and understand that uncertainty is common and acceptable.

## Introduction

Anxiety is the most frequently co-occurring mental health concern in autism, with prevalence rates at least five times higher than in the general population (Kerns et al., 2014; South and Rodgers, 2017; Nimmo-Smith et al., 2020). Autistic individuals who have elevated anxiety symptoms often experience more cognitive, social, and emotional difficulties than autistic individuals who are not also anxious (Keefer et al., 2018; McVey et al., 2018). Thus, it is vital to better understand the mechanisms that underlie anxiety symptoms in autism.

Atypical sensory processing is common in autism and in those who are high in autistic traits, with estimates ranging from 69 to 93% prevalence within autistic individuals (Baranek et al., 2007; Robertson and Simmons, 2013; Horder et al., 2014; Green et al., 2015). Atypical sensory processing in autism may include sensory *hyperreponsiveness* compared to non-autistic peers, in which sensory stimuli (such as a noise) are experienced more intensely, and may contribute to sensory defensive behavior (such as covering one’s ears; Green et al., 2015; Bitsika et al., 2020; Bizzell et al., 2020). Sensory *hyporesponsiveness* involves underreactions to the usual sensory environment and may underlie sensory seeking behavior such as peering at objects from different angles for long periods of time (Dunn, 1997; Green et al., 2015).

Dunn’s (2007) model of sensory processing proposes that an interaction of a neurological threshold and self-regulation determine how one responds to a stimulus. Those with low neurological thresholds respond to sensory input more readily than those with a higher threshold. Those with a passive self-regulation strategy respond to sensory input by allowing the sensory stimuli to make them uncomfortable, while those with an active self-regulation strategy may react in a way to minimize contact with a bothersome sensory stimulus. Based on this theory, Dunn (2007) identified four distinct response patterns: sensation seeking (those with high threshold and active self-regulation strategies), sensory avoiding (those with low threshold and active self-regulation strategies), sensory sensitivity (those with low threshold and passive self-regulation strategies), and low registration (those with high threshold and passive self-regulation strategies). According to Dunn (2007), those who are sensation seeking seek out sensory stimulation actively. Those who are sensation avoiding feel threatened by sensory input and attempt to avoid it, often resulting in rigidity and rituals that make them feel safe. Those who are sensory sensitive may react with aggression and frustration to overwhelming sensory stimuli that they feel powerless to control. Finally, those with low registration tend to respond to sensory stimuli less than typical individuals and often disregard sensory stimuli.

The world can be a fear-inducing place for people who struggle with sensory processing. Effective sensory processing is important for managing stress and appropriately assessing dangerous situations (Lübke and Pause, 2015; Soumiya et al., 2016) and contributes to successful emotion regulation (White et al., 2014). Emerging evidence highlights a significant relationship between sensory processing concerns and elevated anxiety symptoms in both non-autistic (Liss et al., 2008; McMahon et al., 2019) and autistic individuals (Wigham et al., 2014; Uljarević et al., 2015; Neil et al., 2016; South and Rodgers, 2017).

A recent review article by South and Rodgers (2017) proposes that one link between sensory processing and anxiety in autism is mediated by *intolerance of uncertainty* (*IU*), a cognitive construct that describes lower thresholds for tolerance of uncertainty and overall difficulty with handling ambiguous or uncertain situations. IU has been shown to be associated with anxiety in both non-autistic (McEvoy and Mahoney, 2012) and autistic samples (Boulter et al., 2014; Keefer et al., 2016; Maisel et al., 2016). Autism traits that reflect a preference for sameness and reduced cognitive flexibility may likewise be associated with IU (Rodgers et al., 2012; Wigham et al., 2014; South and Rodgers, 2017).

It has been suggested that IU is a biological phenomenon and that all people are born with an innate tendency to be intolerant of uncertainty, as it is evolutionarily adaptive to be afraid of the unknown (Brosschot et al., 2016, 2017). According to this model, this genetic stress response is said to be the “default” and it is inhibited by feelings of safety. Thus, intolerance of uncertainty is alleviated in situations in which safety has been learned and when these feelings of safety are not learned, stress and anxiety may follow. Grupe and Nitschke (2011, 2013) suggest that exposure to uncertainty increases negative responses to events, both physically and emotionally, while also decreasing our ability to avoid the event or to mitigate its potential negative impact.

Most studies in the area of sensory processing and anxiety in autism have focused on child samples until recently. It is important to examine existing models and theories developed for autistic children in autistic adults in order to determine if they are a good fit for autistic people across the lifespan. Autistic adults are vastly understudied, and adults generally have a higher awareness of their internal states, which is useful information for us to examine as we work to better understand the experiences of autistic adults. A recent study by Hwang et al. (2020) has shown that IU mediated the relationship between sensory sensitivity and anxiety and between anxiety and insistence on sameness behaviors in a group of 176 autistic adults. We have previously published data regarding psychophysiological arousal in autistic adults (*n*=31) with both anxious and non-anxious non-autistic comparison groups, which included data from the self-report Adolescent/Adult Sensory Profile (AASP) and an IU measure (Top et al., 2019). Both the autistic and anxious groups showed significant differences from non-anxious controls in several domains of the AASP, while the autism and anxious groups had similar scores on an IU measure. In the current study, we present data from a much larger sample than our prior one, and we use a dimensional approach which allows for in-depth statistical analysis of patterns of sensory processing in autism and its relationship to IU and to anxiety.

**Figure 1:**
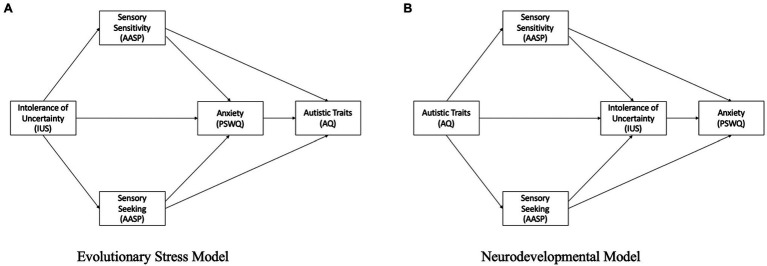
**(A)** Evolutionary stress model **(B)** Neurodevelopmental model.

Previous studies using both child and adult samples have suggested a directional causality from autism traits to intolerance of uncertainty to anxiety. Such models do not easily account for the evolutionary model of learned tolerance for uncertainty suggested by Brosschot et al. (2016, 2017). Based on previous work we anticipate a strong positive association between self-reported autism traits and self-reported anxiety. Our aim is then to test two potential explanations of this association. The first model, which we call the “evolutionary stress uncertainty” model (see Figure 1A) will test the idea intolerance of uncertainty, which is an evolutionary phenomenon common for all people, could explain some of the cognitive aspects of anxiety in autism. This model suggests that intolerance of uncertainty increases sensory sensitivity and sensory seeking behaviors, which then increase feelings of anxiety, which may together contribute to some core autistic traits such as social differences and insistence on sameness. The second model, which we call the “neurodevelopmental” model (see Figure 1B) suggests that neurodevelopmental differences such as sensory sensitivity and a drive for sensory seeking behavior, a preference for sameness, and sticky thinking (i.e., the tendency to get “stuck” on certain thoughts and ruminate on them) with a need to get things “just right,” together underlie a marked increase intolerance of uncertainty which leads to subsequent anxiety.

## Materials and Methods

### Demographics

Archival data were examined for 199 non-autistic adults and 55 autistic adults. Overall, participants ranged in age from 18 to 52, with an average age of 21.8 (± 4.35; 56% male). Within the autism group, ages ranged from 18 to 52, with an average age of 26.2 (± 7.01; 71% male). In the non-autistic group, participants ranged in age from 18 to 27, with an average age of 20.59 (± 1.97; 52% male). Though we provide a demographic breakdown here by diagnosis, we have chosen to run all analyses dimensionally due to the dimensional nature of autistic traits and mental health symptomatology.

### Procedure

Data were aggregated from prior studies conducted by the research team. These prior studies recruited participants through contacting those who agreed to participate in future research studies during prior participation. In order to be included in these studies, participants were required to be verbal and English-speaking and to have an IQ that demonstrated verbal ability within the average range or higher. As the participants included in this study had met these requirements in prior studies, details on their IQ were not collected. Participants in the United States were diagnosed using the ADOS conducted by research-reliable study personnel. Those in the United Kingdom were diagnosed by local clinicians employed by the National Health Service. This study was completed with the approval of the institutional review board at Brigham Young University and with approval from the ethics committee at Newcastle University accordance with the Declaration of Helsinki.

### Measures

#### Autism Traits

The Autism Spectrum Quotient (AQ; Baron-Cohen et al., 2001) is a self-report questionnaire for adults that measures traits associated with autism in five different domains: social skills, attention switching, attention to detail, communication, and imagination. Items are rated on a four-point Likert-type scale ranging from “1 – Definitely agree” to “4 – Definitely disagree.” Scoring is reversed for items for which an agreeing response indicates an autistic trait. The AQ has been shown to have good discriminative validity (Woodbury-Smith et al., 2005) and good interrater and test–retest reliability (Baron-Cohen et al., 2001).

#### Intolerance of Uncertainty

The Intolerance of Uncertainty Scale-12 (IUS-12; Buhr and Dugas, 2002) is a self-report questionnaire that measures the participant’s agreement or disagreement with the idea that uncertainty is unacceptable and leads to frustration, stress, and the inability to take action. Items are rated on a five-point Likert-type scale ranging from “1 – Not at all characteristic of me” to “5 – Entirely characteristic of me” and higher scores indicate a higher level of intolerance of uncertainty. The IUS-12 has been shown to have good construct validity with other similar scales and good test–retest reliability (Buhr and Dugas, 2006).

#### Sensory Sensitivity and Sensory Seeking

The AASP (Brown and Dunn, 2002) is a self-report questionnaire that measures sensory processing in daily life by asking how often the participant engages in a particular behavior within four different domains: low registration, sensation seeking, sensory sensitivity, and sensation avoiding. Items are rated on a five-point Likert-type scale ranging from “1 – Almost never” to “5 – Almost always” and higher scores indicate a higher number of sensory behaviors. The AASP has been shown to have good convergent and discriminant validity as well as good internal consistency and test–retest reliability (Brown and Dunn, 2002). We have elected to use the sensory sensitivity and sensation seeking subscales to measure these two phenomena in our population as separate constructs. These two subscales were selected based on prior research indicating that these are the largest areas of difference between autistic and non-autistic samples (Top et al., 2019), and because in both the literature and in clinical practice these areas are reported by autistic people as the most distressing sensory differences they experience.

#### Anxiety

The Penn State Worry Questionnaire (PSWQ; Meyer et al., 1990) is a self-report questionnaire that assesses the generality, excessiveness, and uncontrollability of worry and anxiety. The PSWQ is considered to be the gold standard for assessing worry. Items are rated on a five-point Likert-type scale ranging from “1 – Not at all typical of me” to “5 – Very typical of me” and higher scores indicate a greater degree of worry and anxiety. The PSWQ has been shown to have good internal consistency and good test–retest reliability (Meyer et al., 1990). One limitation to this study is that the PSWQ focuses on worry as an important but exclusive contributor to anxiety. Beyond that, worry is transdiagnostic and is connected to not only generalized anxiety disorder, but also to social phobia and other anxiety disorders (Einstein, 2014). Worry and intolerance of uncertainty are highly linked, and it has been shown that intolerance of uncertainty is a contributor to anxiety broadly, but especially to worry, making it highly relevant to our analysis and model.

### Data Analytic Plan

As we were using an archival dataset, we excluded any participants from that dataset who were missing any of the measures used in analysis. Additionally, we took a dimensional approach to the measurement of autistic traits and merged all participants into a single group, after first checking for heterogeneity of regression across the samples and found no significant differences. We took this dimensional approach due to the dimensional nature of autistic traits in autistic people and in the general population (Austin, 2005; Kamp-Becker et al., 2010; Ingersoll and Wainer, 2014). We made this choice because we believe that separating our groups would cause us to miss important variance and information that exists along this continuum. We planned two path analysis models for this study to examine the relationships between autistic traits, anxiety, sensory seeking and sensitivity, and IU. The first model tested the idea that intolerance of uncertainty could explain some of the cognitive aspects of autism, as it is a biological phenomenon common in all people. This model suggested that intolerance of uncertainty led to sensory sensitivity and sensory seeking behaviors, which then led to anxiety, and then to autistic traits. The second model suggested that autistic traits led to sensory sensitivity and sensory seeking behaviors, which then led to intolerance of uncertainty, and then to anxiety. This model theorized that autistic traits create a necessary neurodevelopmental context for the development of sensory processing differences, which in turn lead to intolerance of uncertainty and subsequent anxiety.

We used the bootstrapping approach to test significance of indirect effects (Preacher and Hayes, 2008), which is considered appropriate for small sample sizes (MacKinnon et al., 2002). Estimates of indirect effects are based on 5,000 resamples and we report standardized coefficients and bias-corrected confidence intervals for all indirect effects. (A 95% confidence interval not containing zero is statistically significant.) We ran the models using Stata version 16 (StataCorp, 2019).

## Results

**Figure 2:**
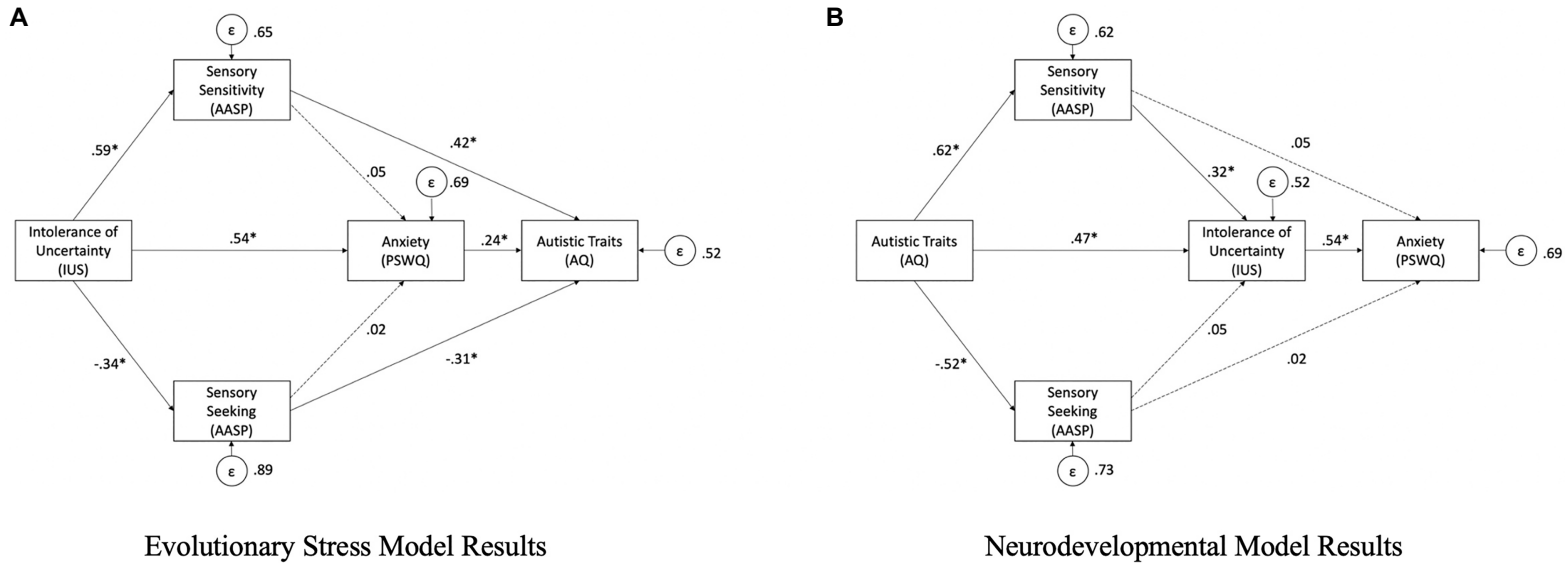
**(A)** Evolutionary stress model results **(B)** Neurodevelopmental model results. Statistically significant pathway (*p < 0.05).

The “evolutionary stress uncertainty” model that places intolerance of uncertainty at the beginning of the model had poor fit, *χ*^2^(2)=31.22, *p*<0.001; RMSEA=0.24; CFI=0.93 (see Figure 2A). Within this model, IU was associated with a significant increase in both anxiety (*b*=0.54, *CI* [0.41, 0.66], *SE*=0.06, *p*<0.001) and sensory sensitivity (*b*=0.59, *CI* [0.51, 0.67], *SE*=0.04, *p*<0.001) as well as a significant decrease in sensory seeking (*b*=−0.34, *CI* [−0.44, −0.23], *SE*=0.05, *p*<0.001). Sensory sensitivity was associated with a significant increase in autistic traits (*b*=0.33, *SE*=0.06, *p*<0.001), but not anxiety (*b*=0.05, *CI* [−0.13, 0.22], *SE*=0.09, *p*=0.60). Sensory seeking was significantly associated with decrease in autistic traits (*b*=−0.31, *CI* [−0.41, −0.20], *SE*=0.05, *p*<0.001), but not in anxiety (*b*=0.02, *CI* [−0.12, 0.17], *SE*=0.08, *p*=0.29). Anxiety was associated with a significant increase in autistic traits (*b*=0.24, *CI* [0.12, 0.35], *SE*=0.06, *p*<0.001).

The “neurodevelopmental” model, with autistic traits at the beginning of the model, had much better fit, *χ*^2^(2)=5.58, *p*=0.06; RMSEA=0.08; CFI=0.99 (see Figure 2B). Within this model, autistic traits were associated with a significant increase in both IU (*b*=0.47, *CI* [0.35, 0.58], *SE*=0.06, *p*<0.001) and sensory sensitivity (*b*=0.62, *CI* [0.53, 0.71], *SE*=*0*.05, *p*<0.001) as well as a significant decrease in sensory seeking (*b*=−0.52, *CI* [−0.60, −0.44], *SE*=0.04, *p*<0.001). Sensory sensitivity was associated with a significant increase in IU (*b*=0.32, *CI* [0.20, 0.43], *SE*=0.06, *p*<0.001), but not anxiety (*b*=0.05, *CI* [−0.13, 0.22], *SE*=0.09, *p*=0.60). Sensory seeking was not significantly associated with either IU (*b*=0.05, *CI* [−0.06, 0.15], *SE*=0.05, *p*=0.38) or anxiety (*b*=0.02, *CI* [−0.12, 0.16], *SE*=0.07, *p*=0.77). IU was associated with a significant increase in anxiety (*b*=0.54, *CI* [0.40, 0.67], *SE*=0.07, *p*<0.001).

## Discussion

The results of this study confirm previous findings that adults who are high in autistic traits tend to have higher levels of anxiety, sensory sensitivity, and IU than non-autistic adults. They also suggest that the neurodevelopmental model for autism and anxiety that has been investigated in our earlier studies (South and Rodgers, 2017) is a better fit for the data than a proposed evolutionary stress model for anxiety in autism. It is important to emphasize that due to the cross-sectional nature of the data used in these analyses, causality cannot be inferred from these models.

Prior research has suggested that sensory processing is one component that contributes to IU. Unique social and emotional profiles in autism may likewise increase IU. Additional support in all these areas may be helpful for decreasing IU and ameliorating subsequent anxiety (White et al., 2018; Rodgers et al., 2019; Costa et al., 2020). The concept of sensitivity to uncertainty has been thought of as an evolutionarily adaptive protective factor, and during typical development most people learn to become more tolerant of uncertainty, but those who do not are more likely to become or remain anxious. However, it appears that in our sample the neurodevelopmental impact of higher levels of autism traits could alter the non-autistic trajectory of learning that uncertainty is common and that it is possible to internalize safety cues and adapt in order to feel safe in the face of uncertainty.

Mental health concerns have been shown to be very disruptive to the quality of life of autistic adults and are likely a critical contributor to extremely high rates of suicidal thoughts and behaviors and death by suicide in autistic adults (Kõlves et al., 2021; South et al., 2021). While anxiety is perhaps the most common mental health problem, standard treatments developed for non-autistic individuals have not had the same levels of reported experiences of effectiveness for autistic people (Crane et al., 2019). Understanding the underlying elements of anxiety in autistic people is critical for understanding how to better intervene. IU appears to be a useful target for intervention, but the research in this area is still developing and exploring what pathways influence the development of both IU and anxiety can help us to develop better interventions. New treatments that have been developed that target IU and underlying components, such as Coping with Uncertainty in Everyday Situations (CUES) by Rodgers et al. (2018, 2019), have shown the promising results for feasibility and preliminary effectiveness for use with autistic people who experience anxiety. Additionally, treatments targeting better management of the sensory world could ameliorate some of the impact of intense sensory processing styles on IU and subsequent anxiety in autistic people. Early occupational therapy focusing on how people interact with and process their sensory environment could be a route to developing better management ability.

### Strengths and Limitations

Our study builds on previous research with a larger sample size and a full measure of sensory processing strengths and difficulties. A real strength of the design is our use of a dimensional definition using autism traits in order to get a full picture of sensory features in our sample. Adults have been overlooked in sensory processing research in autism until recently despite their ongoing sensory concerns, and this study, along with that of Hwang et al. (2020), helps to close that gap. In response to study limitations, future studies could improve on this work by including broader measures of anxiety symptoms, by using observer reports to supplement self-report data, and by comparing other clinical samples, such as clinically anxious samples, to investigate the overlap of anxiety and autism. We acknowledge the lack of mental health measures that have been validated for use in autism samples and that interpretation of our findings is tempered by this ongoing limitation (Hollocks et al., 2019; Howe et al., 2020). Research in this field would benefit from the use of longitudinal data across the lifespan, in order to better examine the trajectory of the development of anxiety in this population.

In summary, while anxiety is a frequent and major concern for autistic adults, typical anxiety treatments have shown less success in this population. Understanding the mechanisms that contribute to anxiety in autistic people is critical for developing better treatments for them. This study supports ongoing research for a model of intolerance of uncertainty as a downstream consequence of intense sensory processing styles in autism (Boulter et al., 2014; Wigham et al., 2014; Maisel et al., 2016; Neil et al., 2016). Both researchers and clinicians should account for sensory processing differences and IU when trying to understand anxiety in autism and create more effective treatments to support autistic individuals with anxiety.

## Data Availability Statement

The raw data supporting the conclusions of this article will be made available by the authors, without undue reservation.

## Ethics Statement

The studies involving human participants were reviewed and approved by Brigham Young University IRB and the ethics committee at Newcastle University. The patients/participants provided their written informed consent to participate in this study.

## Author Contributions

KN-M contributed to data analysis and interpretation and writing the manuscript. NR and DT contributed to conceptualizing the project, data collection, and data analysis. MF and JR contributed to conceptualizing the project and data interpretation. MS contributed to conceptualization, data collection, analysis, interpretation, and writing the manuscript. All authors reviewed and edited the manuscript.

## Conflict of Interest

The authors declare that the research was conducted in the absence of any commercial or financial relationships that could be construed as a potential conflict of interest.

## Publisher’s Note

All claims expressed in this article are solely those of the authors and do not necessarily represent those of their affiliated organizations, or those of the publisher, the editors and the reviewers. Any product that may be evaluated in this article, or claim that may be made by its manufacturer, is not guaranteed or endorsed by the publisher.
